# MLC‐based penumbra softener of EDW borders to reduce junction inhomogeneities

**DOI:** 10.1002/acm2.12082

**Published:** 2017-04-19

**Authors:** Stanislaw Szpala, Kirpal Kohli

**Affiliations:** ^1^ BC Cancer Agency Fraser Valley Centre Surrey BC Canada V3V 1Z2

**Keywords:** dose homogeneity, enhanced dynamic wedge, feathered junction, penumbra modifier

## Abstract

Junctions of fields are known to be susceptible to developing cold or hot spots in the presence of even small geometrical misalignments. Reduction of these dose inhomogeneities can be accomplished through decreasing the dose gradients in the penumbra, but currently it cannot be done for enhanced dynamic wedges (EDW). An MLC‐based penumbra softener was developed in the developer mode of TrueBeam linacs to reduce dose gradients across the side border of EDWs. The movement of each leaf was individually synchronized with the movement of the dynamic Y jaw to soften the penumbra in the same manner along the entire field border, in spite of the presence of the dose gradient of the EDW. Junction homogeneity upon field misalignment for side‐matched EDWs was examined with the MV imager. The fluence inhomogeneities were reduced from about 30% per mm of shift of the field borders for the conventional EDW to about 2% per mm for the softened‐penumbra plan. The junction in a four‐field monoisocentric breast plan delivered to the Rando phantom was assessed with film. The dose inhomogeneities across the junction in the superior‐inferior direction were reduced from about 20% to 25% per mm for the conventional fields to about 5% per mm. The dose near the softened junction of the breast plan with no shifts did not deviate from the conventional plan by more than about 4%. The newly‐developed softened‐penumbra junction of EDW (and/or open) fields was shown to reduce sensitivity to misalignments without increasing complexity of the planning or delivery. This methodology needs to be adopted by the manufacturers for clinical use.

## 1 Introduction

Presence of a junction of fields inside a planning target volume (PTV) is undesired due to formation of a dose peak or dip upon even small misalignment of the fields forming the junction. Similarly, a misalignment‐caused dose peak in an organ at risk (OAR) should be avoided too. The sensitivity to such misalignment can be reduced by softening the dose gradient in the penumbra region. While a steep penumbra is normally desired, it cannot be easily softened on a typical linac to smooth out the junction. Clinical consequences of setup and machine uncertainties on the dose distribution in the junction region were described, e.g., by Holupka et al.^1^ and Rosenthal et al.^2^ The impact on the dose volume histograms (DVH) of the uncertainties of the jaw positions in the junction in the four‐fields monoisocentric breast radiotherapy was discussed recently by Hedin et al.^3^

Fraas et al. utilized a match‐line physical wedge to soften the gradients in the penumbra of diverging fields forming a junction,^4^ while Sohn et al. used a similar concept (referred to as a physical penumbra modifier) to smooth out heterogeneities of the junction of abutting fields.^5^

Shackford et al. softened the dose gradient of the penumbra by dynamically moving the jaws, and implemented this technique for the open fields in craniospinal treatment.^6^

Hong et al. used overlapping IMRT‐planned regions instead of a side‐by‐side matching of diverging fields.^7^

Yom et al. employed field‐in‐field homogenization together with interfraction isocenter shifts to homogenize the dose distribution at the junction of diverging fields.^8^

Application of the feathering concept to craniospinal irradiation by using three isocenters without beam edge matching was described by Cao et al.^9^

Duan et al. described a technique of smoothing the junction between an open field and IMRT fields.^10^ A gentle gradient in the open field was obtained with MLC leaves moving at constant speed across the junction, and the IMRT plan on the other side of the junction was optimized with a baseline dose distribution from the open field described above.

Zeng et al.^11^ extended the technique of Duan et al.^10^ to facilitate use of IMRT only, without a need for (nominally) static fields. The IMRT fields on one side of the (slightly overlapping fields) junction were optimized first using dose objectives with the doses stepped between 80% and 20%, followed by the entire‐region optimization with the fields from the first optimization used as the base plan.

Garcia et al. applied the concept of stepped doses in sub‐PTVs to longitudinally adjacent PTVs treated with helical TomoTherapy.^12^

Wu et al. split the desired intensity (generated through optimization) in a large IMRT plan into two (or more) slightly overlapping intensity distributions such that the intensity in the junction area would be gradually reduced to zero in each region, and used the leaf sequence generator to obtain the leaf sequences.^13^

The techniques described above add to the complexity of the plans. Moreover, most of them cannot be applied to junctions of fields employing enhanced dynamic wedges (EDW).^14^ While inverse planning is often preferred, it is not always possible or practical. In some cases, 3D conformal radiation therapy (3DCRT) is practically as good as IMRT or VMAT, but allows reducing the monitor units (MU) and reducing the dose to healthy tissue.^15^

In this paper we describe development of an MLC‐based penumbra softener for 3DCRT that can be used concurrently with delivery of a combination of EDW and open fields. This design is an extension of the idea of a physical penumbra modifier, but we employ the MLC to dynamically modify the fluence instead of using a physical modifier. Use of the MLC instead of a physical modifier eliminates the need to mount the modifier at the gantry when softer penumbra is desired. The proposed solution was designed to restrict the magnitude of the dose dip or peak at a junction under 1 mm misalignment to no more than about 5%. This design goal was loosely based on the ICRU 50 recommendation to keep the dose in the PTV within 95% and 107% of the prescribed dose,^16^ and the TG142 recommendation for the jaw position indicators, as well as the gantry or the collimator rotation isocenter being accurate within ±1 mm.^17^

## 2 Materials and methods

The softened penumbra fields and the conventional fields (except for the conventional fields used in the four‐field breast plan) were delivered in the developer mode on a Varian TrueBeam linac, version 2.0 MR1, with Millennium 120 MLC (with 0.5 cm wide leaves near the center and 1 cm wide leaves elsewhere). The instructions for moving the MLC leaves and the jaws during beam delivery, as well as instructions for the MV imager, were encoded into xml scripts using in‐house written Matlab scripts, and executed in the developer mode. The conventional clinical‐like plans were planned with Varian Eclipse, and delivered in clinical mode of the TrueBeam linac.

### 2.A. The design

**Figure 1:**
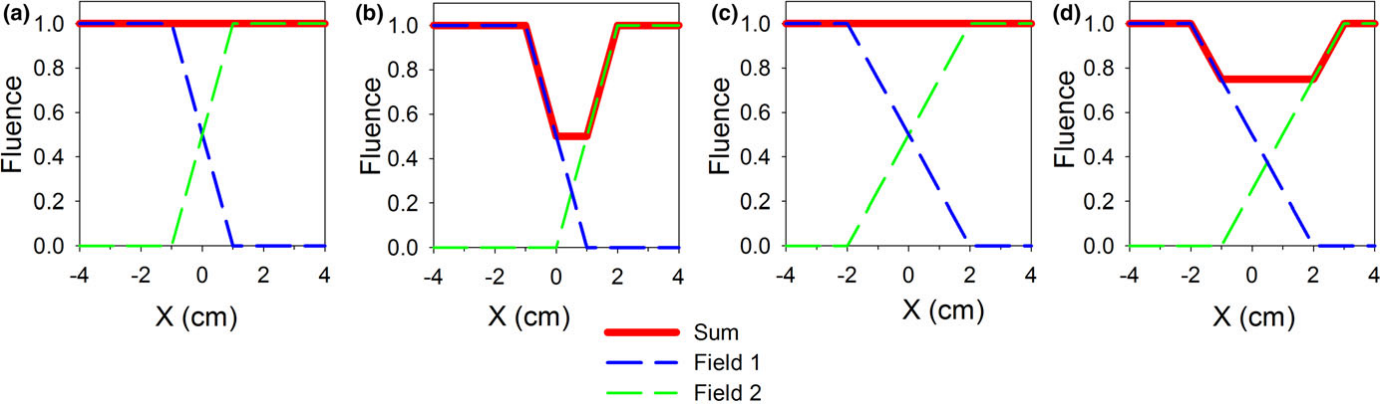
A fluence dip (or a spike; not shown) across the junction of two slightly misaligned fields can be reduced through softening the penumbra of the individual fields. (a) An ideal junction of fields with 2 cm penumbra. (b) The same junction after moving field 2 away from the junction by 1 cm. (c) An ideal junction for fields with 4 cm penumbra. (d) The junction for fields with 4 cm penumbra after the same shift of field 2 as in (b), i.e., 1 cm.

**Figure 2:**
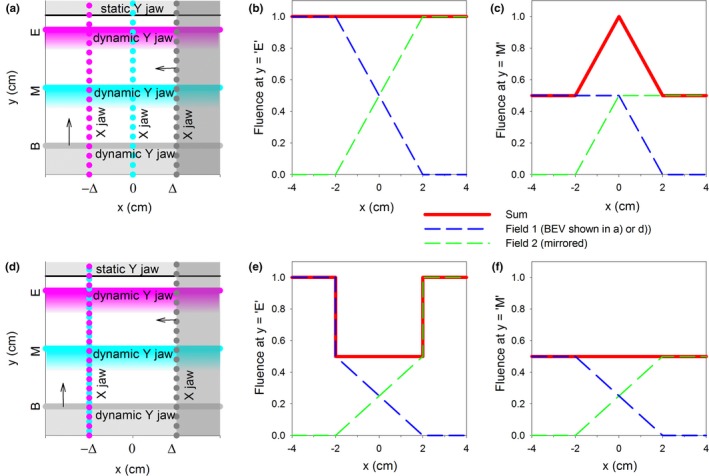
The BEV and the junction profiles of side‐matched EDWs with penumbra widening employing dynamic movement of the X jaw. (a) The BEV showing location of the dynamic Y jaw at the beginning (B), middle (M) and ending (E) of its movement together with the corresponding locations of the X jaw (dotted lines, color coded to match the locations of the dynamic Y jaw), such that a uniform junction (shown in (b)) is formed at *y* = ‘*E*’, but a non‐uniform one (shown in (c)) at *y* = ‘*M*’. (d): The BEV showing movement of the X jaw synchronized with the dynamic Y jaw such that the profile (shown in (f)) at *y* = ‘*M*’ is flat, but is non‐uniform at *y* = ‘*E*’ (shown in (e)).

In a typical junction of static fields, reduction of sensitivity to misalignment from the nominal position (where the positions of 50% fluence coincide for both fields) can be accomplished through widening the penumbra of the individual fields. This is illustrated in Fig. 1 for the given amount of misalignment of one of the fields, doubling the width of the penumbra reduces the dip in the dose by a factor of two. For static fields for which jaws are used as the field borders facing the junction, increasing the penumbra width to the value of *2Δ* can be achieved by moving (at constant velocity) the jaw forming the field border at the junction from *x*_*0*_ − *Δ* to *x*_*0*_ + *Δ*, where *x*_*0*_ is the center of the junction. This strategy does not work for side matching of EDW fields. The problem is explained in Fig. 2 for the junction at *x* = *0*. Initially, the X jaw is retracted from the field by *Δ*. The X jaw starts moving into the field as the dynamic Y jaw (from EDW) begins closing, and the X jaw completes the movement at *x* = −*Δ* when the dynamic Y jaw completes its movement at *y* = ‘*E*’. The corresponding fluence profile across the x direction at *y* = ‘*E*’ is shown in Fig. 2(b) together with the fluence profile of the mirrored field that forms the junction, as well as the sum of the fluences. The summed‐fluence profile is flat, i.e., a perfect junction is obtained. Unfortunately, the summed fluence profile is not flat at other *y* positions, and an example of a highly non‐uniform summed profile is shown in Fig. 2(c) for *y* = ‘*M*’. A triangular peak develops in the summed fluence because after the Y jaw passes beyond *y* = ‘*M*’, no more fluence is added to this profile. To obtain a flat summed profile at *y* = ‘*M*’, one could adjust movement of the X jaw such that its movement is completed at *−Δ* when the Y jaw reaches *y* = ‘*M*’, instead of when the dynamic Y jaw completes its movement, see Fig. 2(d) and 2(f). But in this case the summed‐fields fluence profile at *y* = ‘*E*’ is no longer flat, because after passing *y* = ‘*M*’, the remaining fluence is delivered only outside of the penumbra‐widening zone, see Fig. 2(e).

To avoid non‐uniformities in the summed fluence profile as in Figs. 2(c) and 2(e), the X collimator needs to be modified. To obtain *2Δ*‐wide penumbra centered at *x = 0* for any value of *y* (let's call it *y*_*i*_), the portion of the X collimator at *y = y*_*i*_ needs to move from *x = Δ* to *−Δ*, but at a modified time sequence. Movement of this portion of the X collimator needs to be completed precisely at the instant *t*_*i*_ when the dynamic Y jaw begins to over‐shadow it. Considering *t*_*i*_ depends on position *y*_*i*_, to provide independence of the penumbra width on *y*_*i*_, velocity, *v*_*i*_, of movement of each portion of the X collimator must be:

(1) $$v_{i}\left(y_{i}\right)=2\Delta /t_{i}\left(y_{i}\right)$$

**Figure 3:**
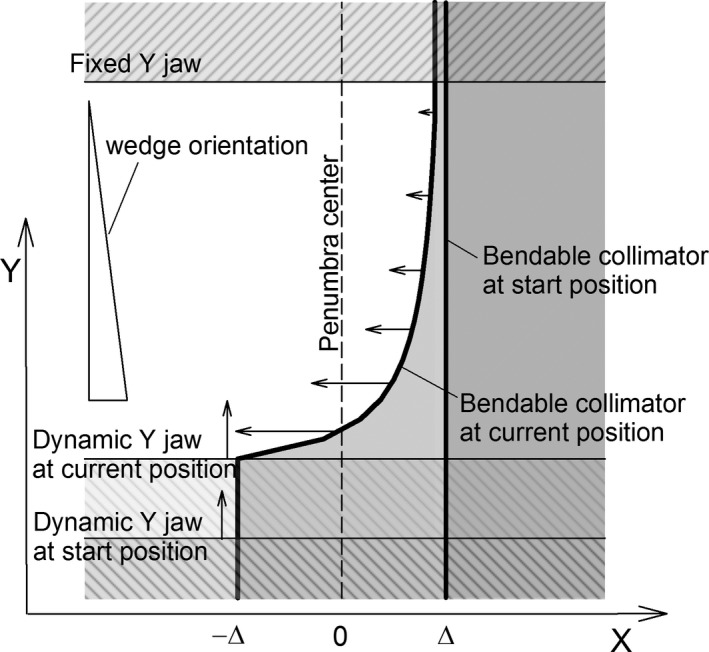
A snapshot of the BEV during the sequence showing use of a bendable collimator to widen the penumbra (to the value of 2∆) at an X border of an EDW field. The portions of the collimator in the shadow of the dynamic Y jaw (in the current position) have already completed their movement from ∆ to −∆, while the remaining portions keep moving (the length of the arrows indicates the velocity).

This necessitates dynamic bending of the X collimator, see Fig. 3.

**Figure 4:**
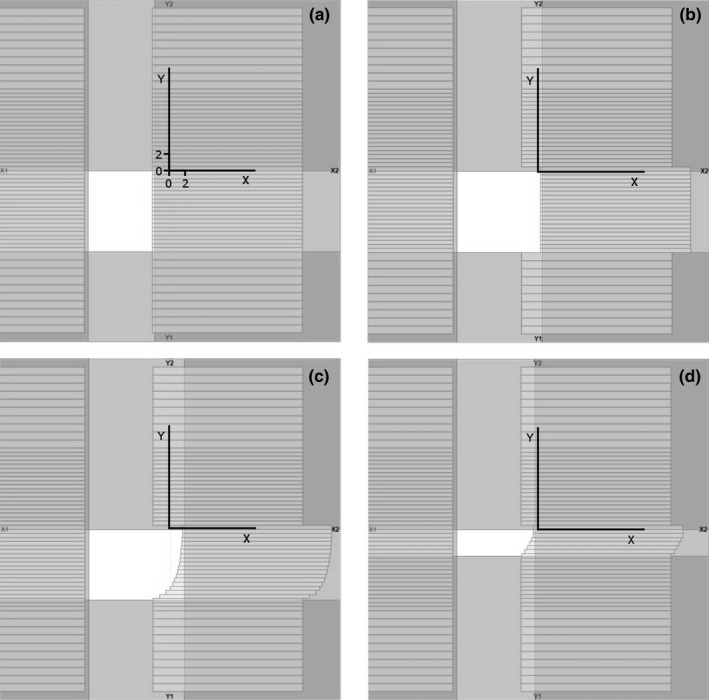
The sequence of leaf movement for 60° EDW Y1‐in with the penumbra softened along x = 0 cm to the width of 4 cm for the nominal field size of Y1 (dynamic) = 10 cm, Y2 = 0 cm, X1 = 10 cm and X2 = 0 cm. (a) The initial positions, (b) the middle of the open‐beam phase, (c) and (d) near the start and near the ending of the dynamic phase, respectively.

**Figure 5:**
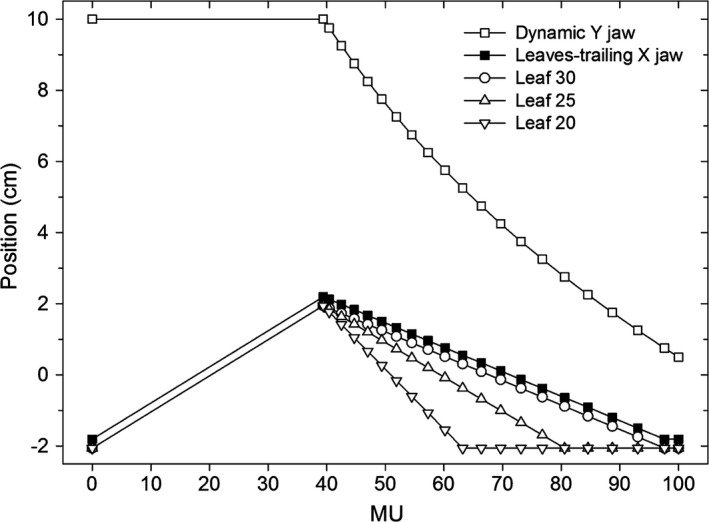
The positions vs. MU for selected leaves, the dynamic Y jaw and the X jaw trailing behind the moving leaves for the sequence of the softened‐penumbra EDW depicted in Fig. 4 (for 100 MU). The symbols are plotted at the MUs corresponding to the control points employed in the sequence.

We obtained the dynamic bending effect by employing the MLC. As in the conventional EDW,^14^ the movement is split into two phases: the open‐beam phase and the dynamic phase where the dynamic Y jaw gradually moves towards the stationary Y jaw. Added in both phases is dynamic movement of the leaves from the MLC bank at the field border that is to be softened. The example below describes use of the dynamically bending collimator added to 60° EDW for 100 MU with Y_dynamic_ = 10 cm and Y_static_ = 0 cm, to obtain 4 cm wide penumbra at the junction at *x* = 0 cm. The open‐beam phase begins with all leaves (from the bank at the softened penumbra) over‐traveling the junction center by the half‐width of the penumbra, *Δ,* equal 2 cm, see Fig. 4(a). Upon turning the beam on, all leaves from this bank begin retracting at the constant velocity, *c*_*0*_, until reaching *Δ* beyond *x* = 0 cm. This means:

(2) $$c_{0}=2\Delta /MU_{0,}$$

where the open phase ends at *MU*_*0*_, and velocity *c*_*0*_ of the leaf is expressed in the units of cm/MU, not cm/s to reflect independence of the delivered MUs on the repetition rate (a convention used in the developer mode). A snapshot of the leaf positions in the middle of the open‐beam phase is shown in Fig. 4(b). The positions of selected leaves are plotted vs. MUs in Fig. 5. The open‐beam phase in this example ends at 39 MU (which is the same as for the conventional EDW of this example). At the start of the dynamic phase, all leaves from the penumbra‐softening bank begin closing the aperture, see Figs. 4(c) and 4(d). While all leaves will finish their movement at the junction center minus *Δ* (the start position of the entire sequence), they will not complete the movement simultaneously. As explained earlier, each section of the bendable collimator, i.e., each leaf, needs to move from *x = Δ* at the start of the dynamic phase to *x = −Δ* at the instant when the dynamic Y jaw over‐shadows the leaf. To accomplish this, the velocity *c*_*i*_ of leaf *i* should be:

(3) $$c_{i}=2\Delta /\left(MU_{i}-MU_{0}\right),$$

where the dynamic phase begins at *MU*_*0*_ and the dynamic Y jaw overshadows the leaf *i* at *MU*_*i*_. This is illustrated in Fig. 4(c): the leaves at *y* = *−*9.75 cm, *−*9.25 cm and *−*8.75 cm have completed their movement (after being over‐shadowed by the Y1 jaw) and the leaves at *y* > *−*8.75 cm continue traveling to their final destination. In both phases the X2 jaw trails 0.25 cm behind the slowest‐moving leaf in order to reduce the inter‐leaf leakage.

While the main concept is explained above, below are additional details:Because the width of each leaf is non‐zero, the MU when the dynamic Y jaw is over‐shadowing a given leaf is not uniquely defined. We defined such MU when the dynamic Y jaw meets the center of the leaf.

The control points governing movement of the dynamic Y jaw, which are defined in the segmented treatment tables (STT) for each wedge, are not appropriate for implementation of the algorithm described above. Instead, we defined the control points at the MUs, for which the dynamic Y jaw meets the centers of the leaves, as seen in the beam's eye view (BEV). These MUs were linearly interpolated from the values in the STT.

By design of the EDW, the dynamic Y jaw stops movement 0.5 cm from the stationary jaw. As the dynamic Y jaw cannot reach the center of the leaf in this 0.5 cm gap, movement of that leaf was chosen to mimic movement of the neighboring leaf (on the dynamic Y jaw side). The positions of the leaves permanently shadowed by the Y jaws are not important, and we set all leaves permanently shadowed by the Y jaws to the initial position of all leaves.

For nominally static (non‐wedged) fields, the penumbra softening may be achieved by moving all leaves at a constant speed as a uniform front (e.g., from 2 cm from the middle of the junction outside of the field to 2 cm from the middle of the junction inside the field). This is similar to the open phase of the softened‐penumbra EDW, and similarly, the X jaw trails 0.25 cm behind the leaves.

Due to the effects associated with the rounded endings of the leaves, it is necessary to add an offset to the leaf positions in order to optimize fluence homogeneity across the junctions. The method of adjusting this offset is described in section 2.B.

### 2.B. Tuning and basic testing with the MV imager

The value of the offset added uniformly to all leaf positions (see section 2.A.) was adjusted to match the fluence at the junction (at *x* = 0 cm) formed by two softened‐penumbra fields to the corresponding fluence in the combined junction‐free field. The time‐integrated fluence was measured with the MV imager collecting data in the “dosimetry” mode. The imager was placed at its center position. The two image halves of the junction image were collected one after another without interrupting image acquisition to avoid uncertainties associated with background subtraction required prior to summing images. The fields corresponding to the largest available opening of the Y jaws during delivery of EDW were used: Y1 = 20 cm and Y2 = 10 cm. 100 MU were delivered for each field at 6 MV, 400 MU/min. The field size in the X direction of each half image was limited to ± 10 cm from the central axis (CAX), and the collimator was set to 270°. Open beams and EDW 60°, Y1‐in, were analyzed.

To compare the sensitivity to misalignments across the junction between the softened‐penumbra and the conventional junctions of 60° EDWs, the corresponding fluences were measured with the MV imager. The misalignments (shifts between −3 mm and 3 mm) were simulated by moving the imager longitudinally between the first and the second half‐image. The moves of the imager were verified to be accurate within no more than 0.25 mm within the applicable range of movement. The same EDW fields were used as during the adjustment of the offset of the leaves.

The fluence profiles were read from the DICOM images from the MV imager using ImageJ 1.43 u (Wayne Rasband, National Institute of Health, USA, http://rsb.info.nih.gov/ij). To improve precision, each profile point was averaged along the junction within ±1 mm from the nominal position, and ±0.4 mm across the junction.

### 2.C. Validation with a four‐field monoisocentric breast treatment using Rando

**Figure 6:**
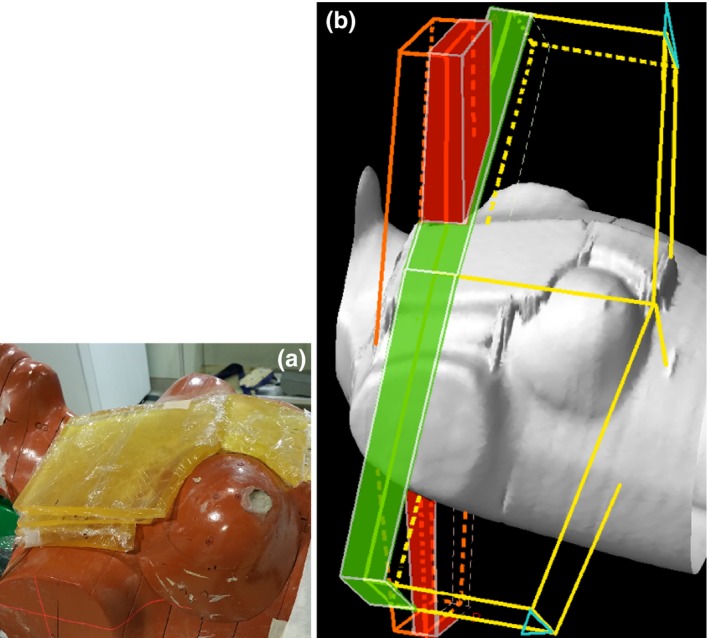
(a) Rando phantom with added layers of superflab was used to evaluate junction homogeneity upon geometrical misalignments in the four‐field monoisocentric right breast plan. Film was placed in the junction region between or underneath the layers of superflab. (b) The fields used in the plans. The tangents of the conventional plan (the subfields are omitted for clarity) are drawn in yellow, while the nodal fields in orange. The orientation of the EDWs of the tangents is shown in cyan. The fields used in the softened‐penumbra plan are similar, except for the softened‐penumbra zones (shown in green for the tangents and in red for the nodal fields).

The performance with the softened penumbra was examined in a four‐field monoisocentric (right) breast plan delivered to a modified Rando phantom (Radiology Support Device, Inc, Ramsey, NJ). EBT3 film (International Specialty Products, Wayne, NJ, currently Ashland, KY) was used to measure the dose distribution in the junction region. The phantom, with right breast attached, was placed on the breast board (tilted by 13°), and was wrapped with superflab layers to facilitate placement of film in approximately coronal plane at the depth of 1.5 cm and 3.0 cm, see Fig. 6(a). Films were exposed for both the conventional plan and the plan with softened‐penumbra junction. The phantom together with the superflab was CT‐scanned, and planned to the dose of 4250 cGy in 16 fractions for the tangents, and 4000 cGy in 16 fractions to the nodes. The monoisocenter was placed 5.0 cm post from the surface of Rando, 7.0 cm right from the midline, and the plane of the junction would touch the inf border of the clavicle. The fields (all 6 MV) used in the conventional plan are described in Table 1, and drawn in Fig. 6(b). The fields used in the softened‐penumbra plan are the same, except the X borders facing the junction are softened within ±2 cm from the nominal field border (for both the open and the wedged fields). In Fig. 6(b), the zones of softened penumbra are drawn in green for the tangents and red for the nodal fields. These zones overlap inside the phantom. Misalignment of the fields for evaluating sensitivity to the isocenter shift was accomplished by shifting the couch superiorly or inferiorly during irradiation of the nodal fields.

**Table 1:** The fields used in the conventional four‐field monoisocentric (right) breast plan delivered to Rando phantom covered anteriorly with layers of superflab to the total thickness of 3 cm. The nominal values in the softened penumbra plan are the same, but the junction‐side X border of every field is softened ±2 cm sup‐inf from the monoisocenter

Field	Gantry (deg)	Collimator (deg)	X1 (cm)	X2 (cm)	Y1 (cm)	Y2 (cm)	Wedge	MU
Med	53	90	18	0	0	13	EDW30out	153
Lat	233	90	18	0	13	0	EDW30in	153
Med sub	53	90	7	0	0	13	None	17
Lat sub	233	90	7	0	13	0	None	17
Ant	0	90	0	5	5	8	None	191
Post	180	90	0	5	8	5	None	34

The film irradiated in the phantom, and the dose calibration film pieces (0, 40, 75, 150, 300, 600 cGy) were scanned 24 hrs after the irradiation with an Epson Perfection V700 Photo flat‐bed scanner (US Epson, Long Beach, CA, USA). The color corrections were disabled, only the red color was analyzed, and 150 dpi resolution was selected. Film orientation with respect to the scanner bed was the same for all scans. The scanner bow‐tie effect along the scanner light source was neutralized by multiplying by a dose‐independent empirically‐determined function (an inverted Gaussian), similarly to Menegotti et al.^18^ For image registration, each piece of film was marked at the perimeter under the shadow of the cross hair. The 2D dose distributions measured with film are evaluated as normalized 2D difference maps: after subtraction of the pixel values, the difference is normalized to the planned dose (in the conventional plan) at the intersection of the CAX with the plane of measurement. The dose profiles were averaged across the profile direction ±2.5 mm to account for influence of the inter‐leaf transmission. With the exception of the profiles of misaligned nodal fields, the dose profiles were measured in separate pieces of film for the combined tangents, combined (normally aligned) nodal fields and all fields summed together (aligned or misaligned). Measuring the profiles for combined fields, instead of numerical addition of the corresponding dose profiles, was meant to avoid film registration inaccuracies influencing the profiles. The individual profiles of the misaligned nodal fields were numerically shifted from the profiles of the properly aligned fields to avoid unintentional shift of the profiles due to uncertainty of the film setup.

## 3 Results

### 3.A. Fluence along the junction

In this section we demonstrate that the fluence in the junction of softened‐penumbra EDW fields and/or open fields closely resembles the fluence of a combined (junction‐free) conventional field. Such data validates that the bendable‐collimator design allows avoiding fluence inhomogeneities along the junction [as seen in Fig. 2(c) and 2(e)] in the profiles across the junction). Failure of this test would render the design clinically unusable.

**Figure 7:**
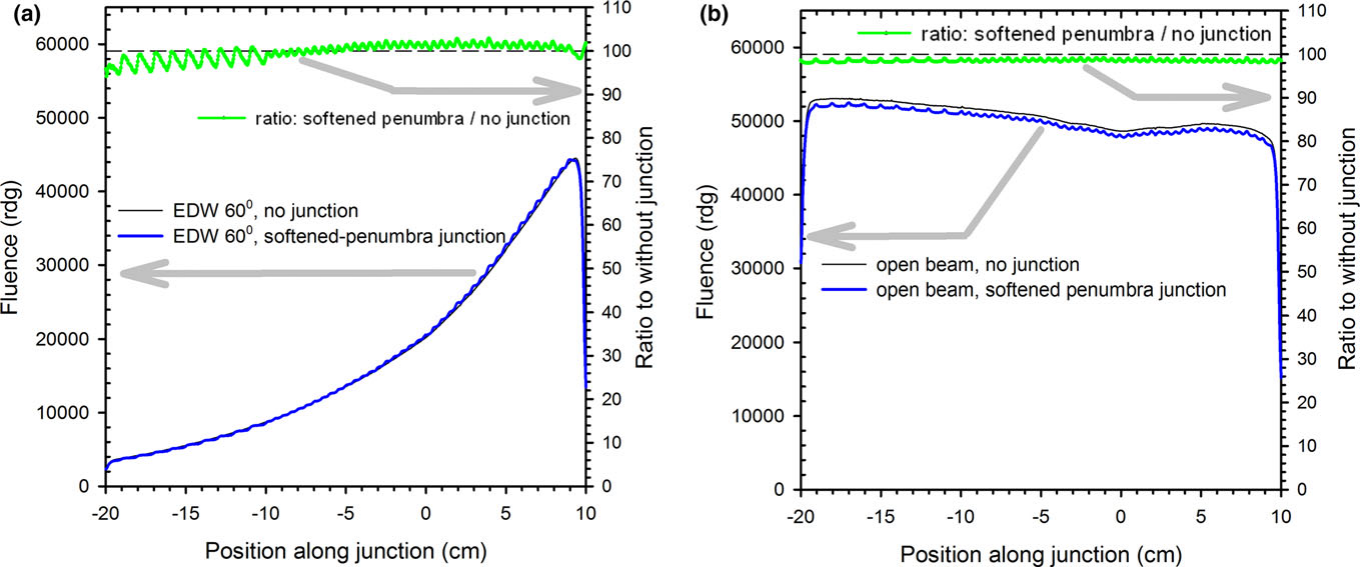
The fluence profiles along the center of the junction (at *x* = 0 cm) of (a) 60°EDWs and (b) open fields for the softened penumbra fields and the corresponding junction‐free fields. The ratios of the softened‐penumbra fields to the junction‐free field are also shown. The leaves offset was set to −0.6 mm (all leaves extended by 0.6 mm) to simultaneously optimize agreement for the junction of the 60˚ EDWs and of the open fields. The dashed lines represent the ratio of 100%.

The fluence profiles along the junction of the softened‐penumbra fields after adjustment of the leaf offset are plotted in Fig. 7. The junction of the 60˚ EDWs and of the open beams are shown, together with the corresponding profiles for the combined field without a junction. The same data was also plotted as ratio curves (for each position along the profile the fluence in the softened‐penumbra junction was divided by the fluence in the combined field). Ideally, the profiles of the softened‐penumbra and the conventional fields should be identical, and only minor discrepancies are seen. The small modulation in the softened penumbra plots is primarily due to the discrete width of the leaves (5 mm or 10 mm wide) of the MLC, and partially due to the inter‐leaf leakage, which is not entirely eliminated by the X jaw trailing behind the MLC bank. The modulation in the profile of the open beams is due to the inter‐leaf leakage. Changing the leaf offset adds or subtracts a small amount of dose to the profiles of the softened‐penumbra junctions, and consequently shifts the ratio curves up or down (not shown). The offset value of −0.6 mm, i.e., the leaves were extended to close the beam aperture by 0.6 mm, was chosen as a compromise between the optimum value for the open‐beam junction and the junction of the 60° EDWs. Because EDWs for other angles are constructed from a combination of an open field and a 60° EDW, all other wedge angles will exhibit discrepancies in‐between the two extremes. The discrepancies are within the range of [−2%, 3%] for regions modulated with 5 mm leaves, and [−5%, 1%] for 10 mm leaves. Such discrepancies are much smaller than the hot and cold spots observed in the conventional junctions upon misalignments of only 1 mm, see section 3.B. The fluence away from the softened‐penumbra junction is practically undistinguishable from the fluence in the junction‐free combined field (not shown).

### 3.B. Fluences at the X1‐X2 junction of two EDWs upon misalignment of fields

In this section we evaluate the design for sensitivity of the summed fluence to field misalignment, and demonstrate improvement over conventional junctions.

**Figure 8:**
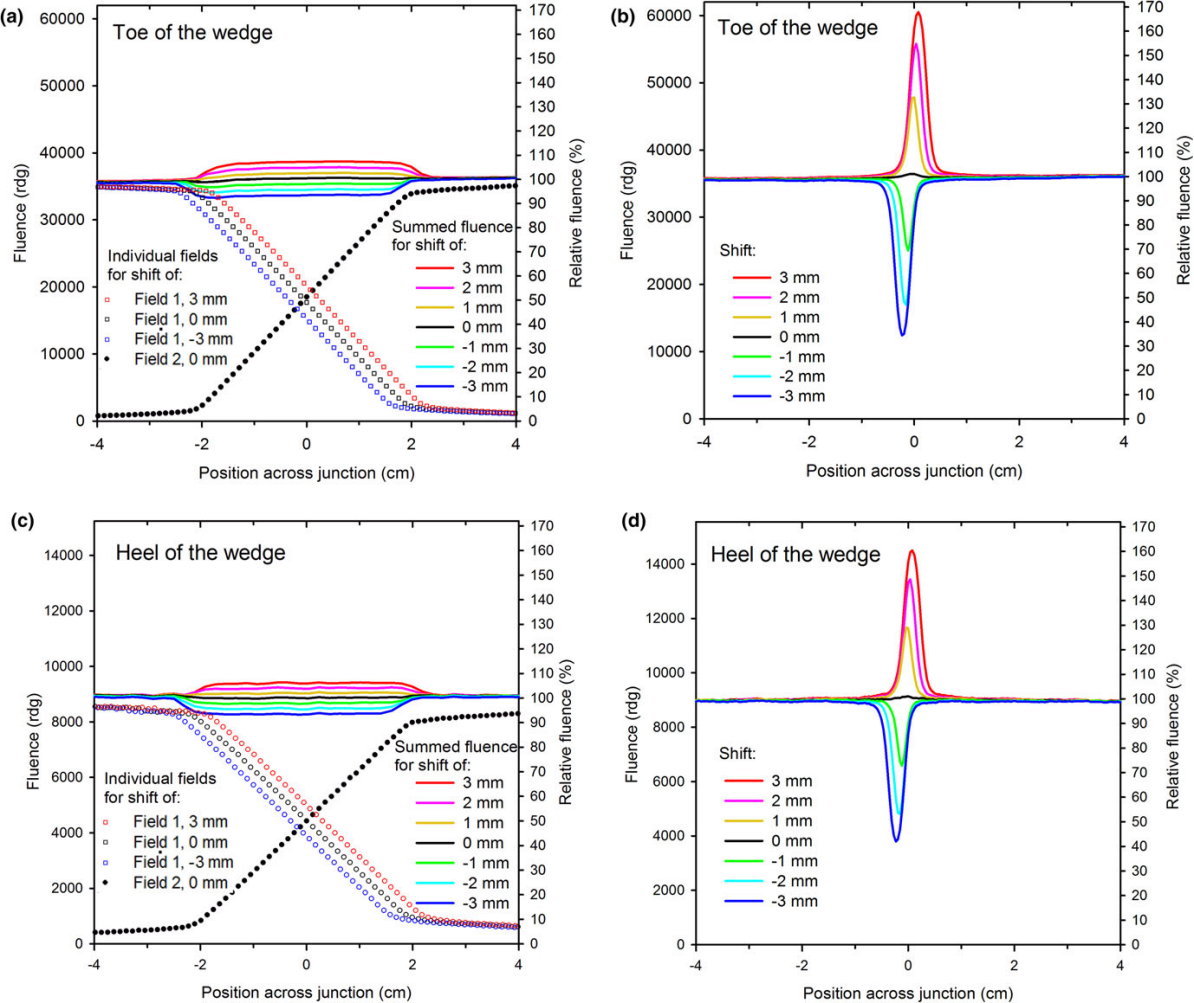
The fluence profiles across the junction of side‐matched 60°EDW fields for properly aligned and misaligned fields. (a) softened‐penumbra fields in the toe region of the wedge, (b) conventional fields, toe region, (c) softened penumbra, heel region, (d) conventional fields, heel region. Selected fluence profiles of the individual fields are also shown (omitted for clarity for the conventional junction). Note the fluences in the toe region are larger than in the heel region, even though the relative fluences are similar.

The summed‐fields fluence profiles (collected with the MV imager) across the junction of side‐matched softened‐penumbra 60° EDWs are plotted in Fig. 8 for various amounts of misalignment of the fields. Also plotted are the fluence profiles of the individual softened‐penumbra fields for selected values of misalignment. The profiles are plotted separately at two different values of the y coordinate to illustrate the performance in the junction region both at the heel and at the toe portion of the EDW. The fluences in the junction region of the misaligned softened‐penumbra fields (from −2 cm to 2 cm) differ only slightly from the junction without any misalignment (Figs. 8(a) and 8(c). The discrepancy increases with the misalignment, but it is only about 2% per each mm of misalignment. In contrast, the discrepancy for the conventional junction is much larger, see Figs. 8(b) and 8(d), and is about 30% for 1 mm of misalignment, and over 60% for 3 mm of misalignment. The relative fluence heterogeneities in the softened‐penumbra junction are similar in the heel and in the toe region of the EDWs.

### 3.C. Junction in the four‐field breast plan

In this section we examine a clinically‐relevant four‐field monoisocentric breast plan. The correspondence of the softened‐penumbra plan to the conventional plan is inspected, as well as sensitivity of the dose distribution to introduced shifts (away and towards the junction) of the fields forming the junctions. Reduction in sensitivity of the dose variations to shifting the fields is demonstrated over conventional junctions.

**Figure 9:**
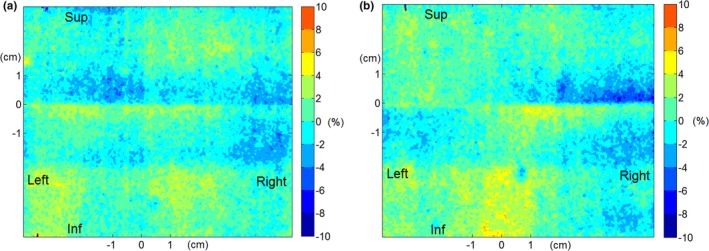
The near‐junction dose difference (measured with film, no misalignment of the fields) between the softened penumbra junction and the conventional junction in the four‐field monoisocentric breast plan (normalized to the planned dose at the intersection of the CAX and the film). The film was placed approximately in the coronal plane at two different depths to examine performance near the heel and near the toe of the EDWs: (a) depth = 1.5 cm, (b) depth = 3 cm. The junction is located at sup‐inf = 0 cm, and the CAX at (0 cm, 0 cm).

The dose distribution of the softened‐penumbra junction does not need to be identical to the distribution in a conventional‐junction plan as long as it meets the planning objectives. Nevertheless, similarity of the two distributions is desired, as it will allow replacing the conventional plans with the softened‐penumbra plans. Similarity of the two plans (in the ideal setup, i.e., without any shifts) is demonstrated in Figs. 9(a) and 9(b) in the near‐junction region. The normalized difference maps between the softened‐penumbra plan and the conventional plan in the approximately coronal plane are plotted at clinically representative depths of 1.5 cm and 3.0 cm. In general, the differences do not exceed about ±4% at either depth, with discrepancies in small regions up to about ±6%. These differences demonstrate lack of artifacts seen in Figs. 2(c) and 2(e).

**Figure 10:**
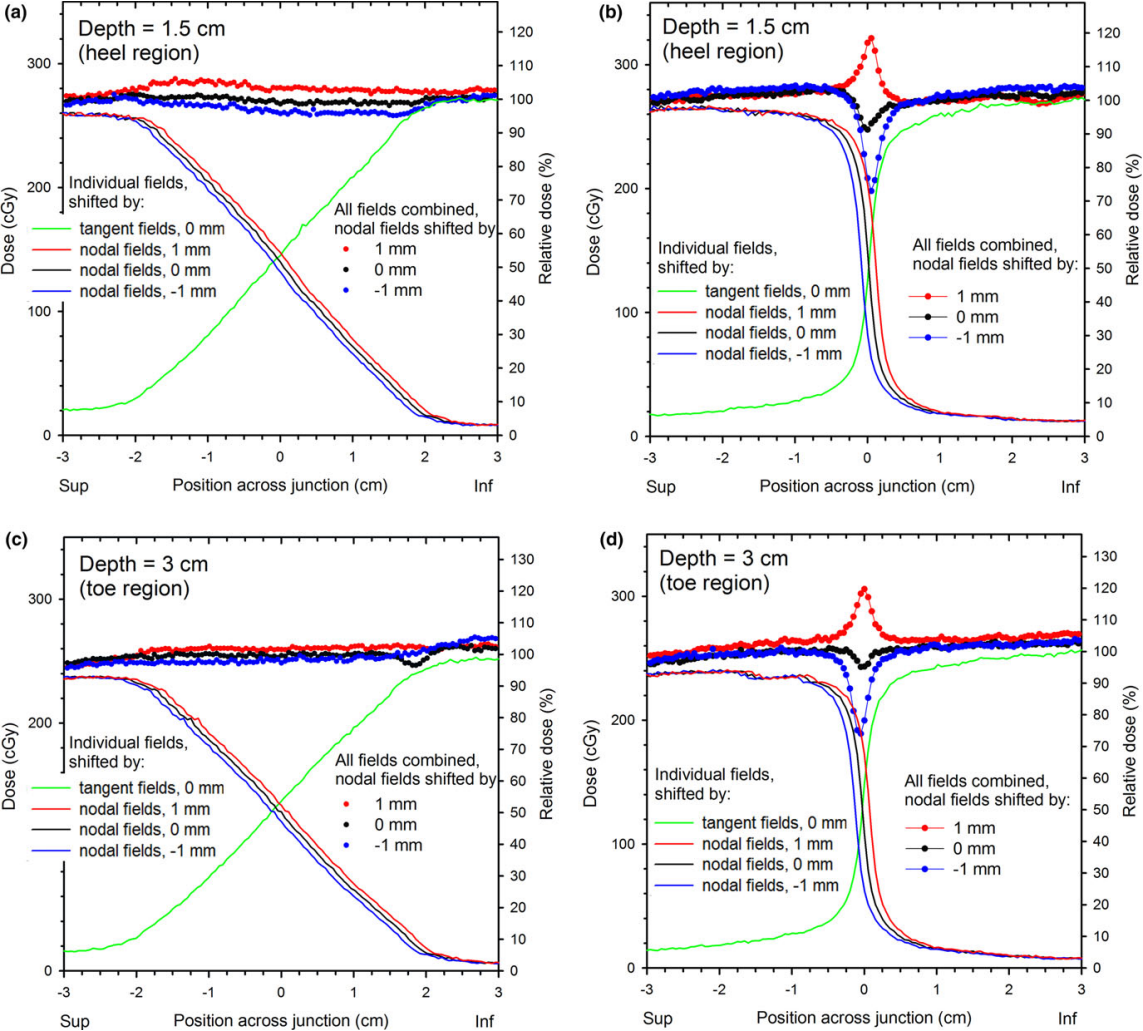
The dose profiles across the junction (at the left‐right = 0 cm) in the softened‐penumbra, (a) and (c), and in the conventional, (b) and (d), four‐field monoisocentric breast plan delivered to the Rando phantom for various amounts of misalignment of the nodal fields. The combined dose profiles are shown together with the profiles of (grouped) individual fields superiorly and inferiorly of the junction. The profiles at the depths of 1.5 cm, (a) and (b), and 3 cm, (c) and (d), are shown separately to illustrate performance near the heel and near the toe of the EDWs.

The improvement in the dose uniformity upon misalignment of the fields in the junction between the nodal fields and the tangents is illustrated in Fig. 10. Plotted are the dose profiles measured with film across the junction without and with shift of the nodal fields 1 mm away or towards the junction. The combined‐fields dose profiles and the (grouped) individual fields are plotted for the softened‐penumbra and the conventional‐junction. The profiles were measured at two depths (1.5 cm and 3 cm) to examine sensitivity to misalignment near the toe and near the heel of the wedge (of the tangent fields). In the softened‐penumbra junction, the dose changes upon misalignment of the fields do not exceed about 5% from the misalignment‐free data at either depth. In the conventional junction, the corresponding effect of the misalignment is about four to five times larger: about 20% peak or 25% dip. The actual values for the conventional junction may be slightly different, as a dip of about 4% or 8% is seen even for nominally aligned fields. These dose irregularities are due to limitations of adjustment of the X jaws and walk of the isocenter upon gantry rotation, and are the reasons why this work was done.

## 4 Discussion

**Figure 11:**
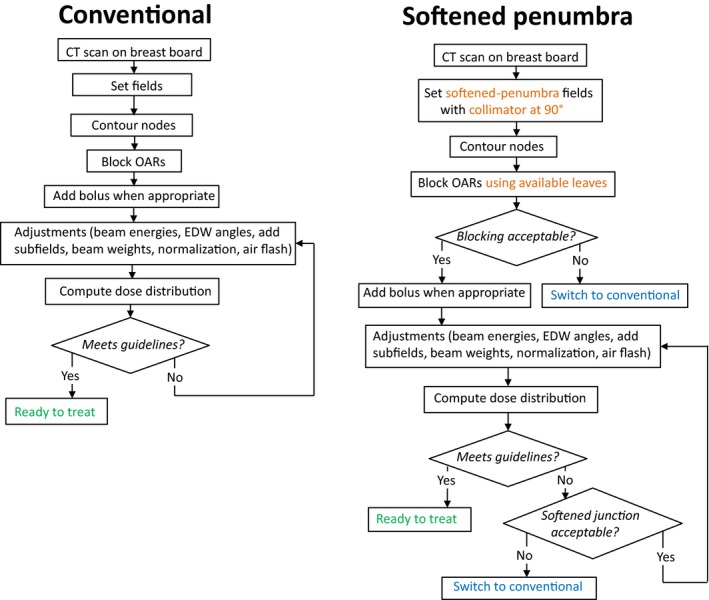
The clinical‐process diagram for the proposed softened penumbra and the conventional four‐field monoisocentric breast plans.

The design described above met the design goal of keeping the dose changes within ±5% upon 1 mm misalignment of the fields forming the junction. As such, the design adds another option to minimize dosimetric consequences of geometrical misalignments of fields forming junctions. It should be noted that in general, this design goal cannot be achieved with conventional junctions, as demonstrated in section 3.C). The design does not significantly increase complexity of the planning nor complexity of the delivery of the treatment, and the beam delivery time is not affected. Once implemented in the treatment planning system, the dose distribution will be available for review, and use of the softened penumbra will be almost transparent to the planner. The main difference between the softened penumbra and the conventional plans is that the planner would select the field borders on which penumbras should be softened, and the calculated dose distribution would be evaluated as normally. An example of a clinical process diagram for the four‐field monoisocentric breast plan highlighting differences between the softened‐penumbra plan and the conventional plan is shown in Fig. 11. The diagrams are similar except the conventional fields are replaced with the softened‐penumbra fields and the collimator rotation is restricted to 90° (or 270°) to facilitate movement of the MLC leaves across the junction. The process reserves an option to switch to the conventional approach.

While the dose distribution across the junction of the softened‐penumbra junction is close to the distribution from a conventional plan, it is not identical. This should not be a problem clinically, as it is the plan with the softened penumbra that is to be evaluated for meeting the plan objectives, including dose heterogeneity inside the PTV within −5% and 7% of the prescribed dose, and absence of hot spots. In some cases, the softened‐penumbra plan may be actually preferred over the conventional plan, even in the absence of geometrical misalignments. This may be the case of four‐field monoisocentric breast plans, where the softened‐penumbra junction feathers out dose gradients in the interpectoral axillary nodes region located in the vicinity of the junction.

Clinical implementation of the softened‐penumbra requires adaptation by the manufacturers of linacs and treatment planning software. In the clinical mode of Varian's TrueBeam v2.0 MR1, concurrent movement of the jaws and the MLC is not allowed. The dose distribution in the presence of simultaneous movement of the dynamic jaw of the softened‐penumbra EDW and the leaves of the MLC can be computed similarly as in the step‐and‐shoot method with the control points matching those in the softened‐penumbra design. Refinement is possible by introducing interpolated control points (needed only during the calculation of the dose distribution, and not during the delivery on the linac).

This design relies on orthogonality of the junction plane to the skin at the beam entrance, and this limitation is shared with many other designs employing penumbra softening. This is because adding the doses from the ramped penumbras of the fields on the opposite sides of the junction yields a flat dose profile only under such orthogonality. This orthogonality requirement is approximately satisfied for four‐field monoisocentric breast plans. The anterior, the posterior and the medial tangent fields typically satisfy the orthogonality requirement, and the lateral tangent field approximately. The latter is true because the ratio of the tissue‐phantom ratios (TPRs) for the depths at the superior‐inferior coordinate varied by ±2 cm is typically no more than a few %. In any case, inspection of the computed dose distribution would allow confirming the absence of artifacts at the junction potentially caused by the design.

Blocking OARs may be partially affected by the design because the leaves of the MLC need to move across the junction, and because the leaves used to soften the penumbra cannot be simultaneously used to block an OAR. In many cases, the leaves at the edge of the field may be used to block an OAR instead of softening the penumbra, without any noticeable influence on the junction. This is the case of blocking the spinal canal in the nodal fields of four‐field monoisocentric breast plans, where the spinal canal is entirely outside of the BEV of the tangent fields. There are no restrictions on blocking OARs using the other bank of the MLC (e.g., the chin or the heart in four‐field monoisocentric breast plans), as those leaves are not employed to soften the penumbra. Regardless, the computed dose distribution should be examined to confirm the plan meets the planning objectives.

The choice of ±2 cm as the width of the softening zone is not unique. Increasing the softening zone allows reducing sensitivity to misalignment of the fields at the junction even further, even though the value of ±2 cm allowed meeting the design goal. On the other hand, it is not recommended to extend the softening zone much beyond ±2 cm. This is in part because widening the penumbra results in enlarging the underdosed zones in the high‐dose portion of the penumbra and the overdosed zones in the low‐dose portion in the regions where the fields across the junction do not overlap. This phenomenon is not unique to this design, and it may also be present in other methods that soften a junction. Extending the width of the softening zone also reduces the maximum usable field length, which is reduced by the half‐width of the zone (2 cm here) from the machine limit (20 cm for half‐blocked fields). Longer fields are possible (up to 38 cm) for non‐half‐blocked fields, but couch rotation would be needed to maintain orthogonality of the junction plane to the skin, as well as subfields. It should be noted the usable field length is not restricted by the range of movement of the MLC carriage (15 cm on Varian linacs), because the leaves move only 4 cm during the sequence.

To eliminate the residual inter‐leaf leakage (not blocked by the trailing X jaw) in the open‐beam phase of the softened‐penumbra EDW or softened‐penumbra nominally static fields, we considered using the X jaw instead of the MLC. Unfortunately, combining such fields into junctions introduced additional artifacts due to differences in the shape of the dose penumbra of a square jaw and round‐ended leaves of the MLC.

While there is a current trend to choose IMRT or VMAT over 3DCRT, there are situations when inverse planning techniques are not practical, and junctions of conventional EDWs and open fields are used. In particular, IMRT will suffer from uncertainties in the regions where the PTV is narrow in the BEV, and the instantaneous leaf gaps are small, in which case accuracy of modeling the rounded‐leaf ends in Eclipse is reduced. Also, due to the presence of the isocenter walk upon gantry or collimator rotation, combining IMRT fields across a junction may be susceptible to dose spikes/dips at the junction when different gantry and/or collimator angles are used for the fields across the junction. Use of VMAT for some sites is arguably not ideal, e.g., for breast. This is because of unnecessary irradiation of the lung from the beam exit when the beam incidence is approximately normal to the skin.

It may be argued that fractionation averages junction uncertainties, which eliminates a need for improving junction quality. This is not always true, because a systematic misalignment will not average out. In addition, there is a growing tendency to reduce the number of fractions, and consequently diminish the averaging effects from the process.

Compensation for organ movement can lead to additional uncertainties at the junctions. This is the case for the junction in four‐field monoisocentric breast plans delivered during deep inhalation breath hold (DIBH). The reason is the nodal fields are delivered on a different breath hold than the tangents, and for practical reasons, the chest position (including the sup‐inf alignment of the body with the CAX) is only held within a range of positions, not at the same position on every breath. The softened‐penumbra design should reduce uncertainties related to organ‐movement.

Softened penumbra field borders might also be considered when disease progresses, and a larger PTV might be needed in the future. This would happen when treating a breast as tangents, with a possibility of treating the axillary nodes in the future: the superior borders of the tangents would be softened out initially, and the nodal fields with softened inferior borders would be added in the future when required. Half‐body irradiation could also benefit from this approach through reduction of junction artifacts upon matching the opposite body half in the future. Ideally, the fields would be delivered under extended source‐to‐skin distance (SSD) with rotated couch to keep the (future) junction in the axial plane, and subfields would be employed to even out dose gradients caused by variations of the SSD. While this approach does not require use of wedges, the penumbra softener developed in this project allows delivery of open fields too.

The softened‐penumbra technique may be also useful in matching long fields employing hinged wedge pairs. A hinged wedge pair offers better control of the dose homogeneity at depth over a single beam or a parallel‐opposed pair, and clinical applications could include leg sarcoma or spinal canal. The softened‐penumbra fields for treatment of the spinal canal could be also matched to the brain fields (conventional or IMRT/VMAT) for complete cranio‐spinal treatment with the junction(s) practically insensitive to misalignments. This would be similar to the solution of Duan et al.,^10^ except for use of hinged wedge pairs to improve control of the dose distribution at depth.

Softened‐penumbra fields might also improve junction quality for junctions that are not geometrically matched, e.g., when large fields are delivered without couch rotation, and the fields are only matched at a single depth, e.g., at the skin.

## 5 Conclusions

A prototype penumbra softener was developed to reduce misalignment‐caused dose heterogeneities at junctions of a side border of EDWs and/or open fields in 3DCRT. This penumbra softener employs the MLC, such that the movement of the leaves is synchronized with movement of the dynamic Y jaw of the EDW. Performance was evaluated in the four field monoisocentric breast plan delivered to a Rando phantom, and approximately four‐fold reduction of the dose dips or spikes in the junction was observed when compared to the plan with the conventional junction. The time required for planning and complexity of the plan with the softened penumbra junction should be similar as for the conventional‐junction plan. The system requires adoption by the manufacturers for clinical applications.

## Conflict of Interest

A provisional patent for the design was filed with USPTO.
